# Colouterine Fistula: A Rare Presentation of Severe Diverticular Disease

**DOI:** 10.7759/cureus.74162

**Published:** 2024-11-21

**Authors:** Jessica M Orgovan, Byron D Dodson

**Affiliations:** 1 Medical School, West Virginia School of Osteopathic Medicine, Lewisburg, USA; 2 General Surgery, Mon Health Medical Center, Morgantown, USA

**Keywords:** colouterine fistula, diverticular fistula, diverticulitis, diverticulosis, pathologic fistula

## Abstract

Colouterine fistula as a sequela of diverticulitis is an extremely rare complication due to the extraordinarily thick layer of myometrium of the uterus. Because of this, an aggressive clinical evaluation is required to rule out other potential causes of fistula formation such as malignancy. However, imaging and laboratory techniques may be inconclusive, and surgery with pathologic analysis may be required for a definitive diagnosis. The case presented here illustrates the atypical presentation of a 63-year-old woman with acutely symptomatic, severely extensive diverticular disease with resultant colouterine fistula. The patient underwent exploratory laparotomy, sigmoidectomy with end colostomy, appendectomy, and total abdominal hysterectomy with bilateral salpingo-oophorectomy. Eventually, she was discharged with an excellent prognosis and had an uneventful recovery. This case is being presented because of the rarity of the disease course as well as the complexity of the decision-making and surgical approach that resulted in patient recovery.

## Introduction

Diverticular disease encompasses a group of conditions in which diverticula form in the colon; this includes diverticulosis, diverticulitis, and diverticular bleeding. Colonic diverticula consist of outpouching of the intestinal wall and are a quite common anatomic alteration found in humans [1]. Diverticulosis has a wide range of severity, with most individuals remaining asymptomatic [1]. However, it is estimated that 25% of those with diverticulosis will develop symptomatic disease [1]. Complications arise when diverticula become inflamed, which can usually be medically managed with antibiotics and lifestyle changes. However, complicated diverticulitis can manifest as abscess formation, fistula formation, perforation, peritonitis, hemorrhage, and sepsis. These complications require hospitalization and potentially surgery [1]. The exact triggers that induce progression from diverticulosis to diverticulitis remain unknown [2]. It is speculated that geographic influences, lifestyle variables, and microbiota of the gastrointestinal tract are some of the factors implicated in diverticular disease, with a higher incidence occurring in the United States and Europe in recent years [2].

When fistulas do occur because of complicated diverticular disease, the most common types are colovesical followed by colovaginal [3]. Colouterine fistulas are exceedingly rare, due to the thickness of the muscular layer of the uterus called the myometrium [3]. A colouterine fistula is much more likely to form as a result of pelvic malignancy, radiation, or obstetric trauma [3]. Colouterine fistulas have been reported to present similarly to colovaginal fistulas, with the most common symptom being foul-smelling, hemorrhagic, purulent, or feculent vaginal discharge. However, symptoms vary widely, ranging from vague abdominal pain to septic shock [4]. There have been few case reports of colouterine fistula in the literature, all demonstrating the rarity, complexity, and challenges in the diagnosis and management [5-13].

## Case presentation

This is the case of a 63-year-old woman who presented to an outside emergency department for worsening dizziness and night sweats that had been ongoing for a month. She also complained of difficulty urinating and feeling as if she was not completely emptying her bladder. She noted that when she strained to try and empty her bladder or pass gas, a liquid stool was coming out from what she believed to be her rectum. She noted that her urine appeared cloudy with a strong odor. She denied any vaginal discharge or bleeding and did not believe she was passing stool via her vagina. She did admit to a few days of watery diarrhea before the onset of her other symptoms but has never had trouble with her bowel habits in the past. She denied any associated nausea/vomiting, abdominal pain or tenderness, bloating, burning with urination, hematuria, or melena. The patient stated she had never had any symptoms like this before nor any history of or treatment for diverticulitis.

The patient has a past medical history significant for hypertension and gastroesophageal reflux disease (GERD), well managed with oral anti-hypertensives and proton pump inhibitors. Her past surgical history consisted of arthroscopy of the knee and a routine screening colonoscopy seven years ago, in which she noted there were no significant findings and was told to follow up in 10 years. Her gynecologic history consisted of being multiparous, with uneventful pregnancies resulting in uncomplicated vaginal deliveries, and being post-menopausal. The patient admitted to a history of breast cancer in her paternal grandmother, colon cancer in her maternal grandmother, and uterine cancer in her mother. The patient denied the use of alcohol, tobacco products, or recreational drugs.

**Figure 1:**
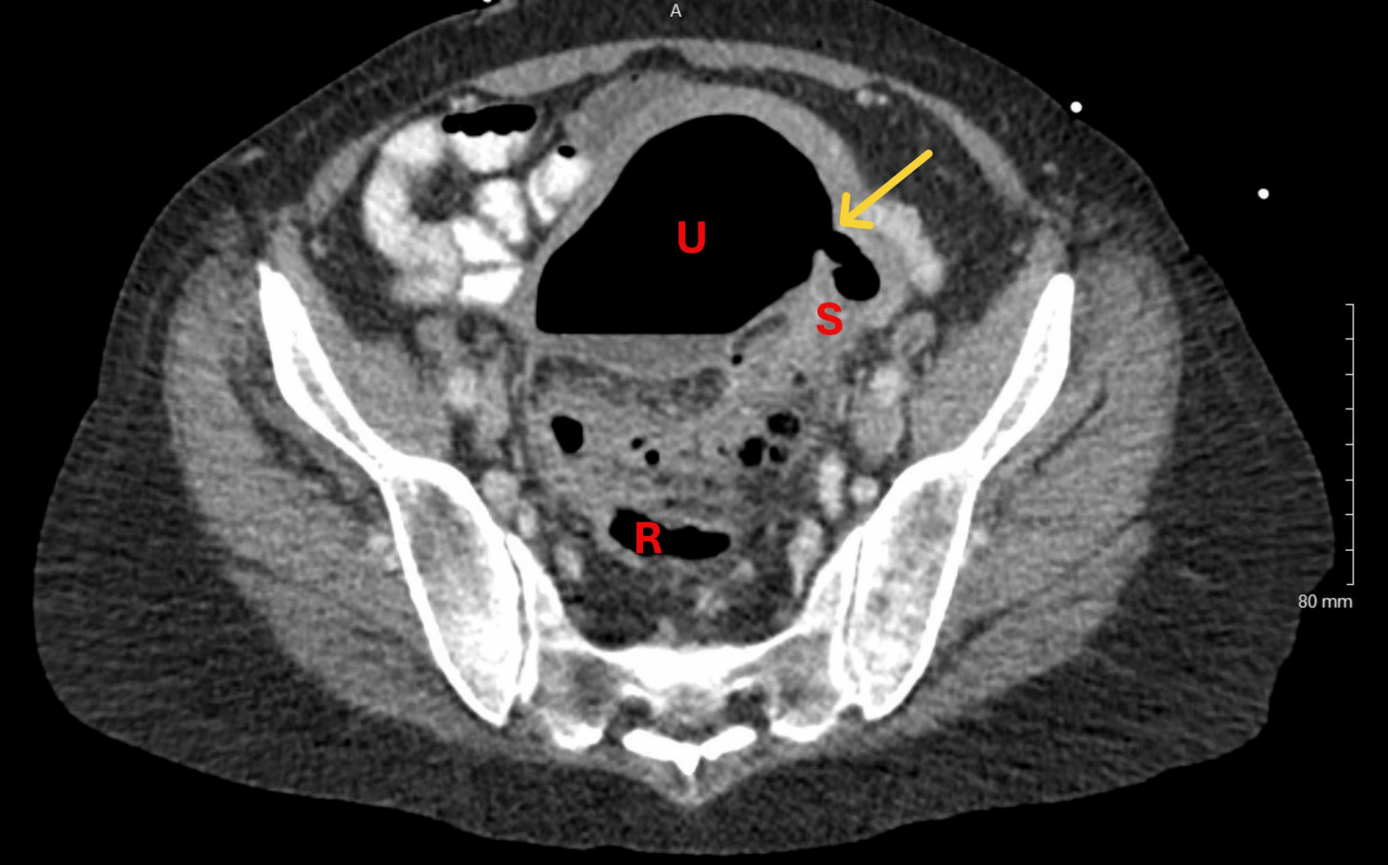
Contrast-enhanced computed tomography (CT) scan of the pelvis, axial view. The fistulous tract (yellow arrow) is shown between the uterus (U) and sigmoid colon (S). Rectum (R) is labeled for reference.

**Figure 2:**
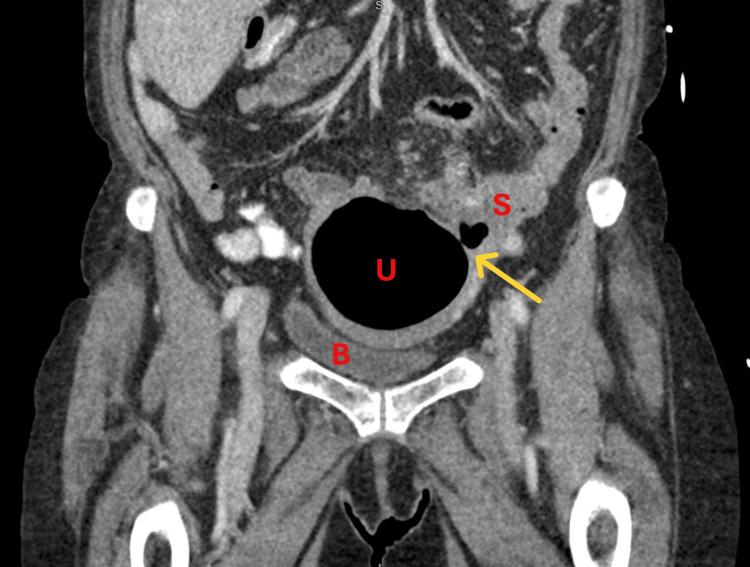
Contrast-enhanced computed tomography (CT) scan of the pelvis, coronal view. The fistulous tract (yellow arrow) is shown between the uterus (U) and sigmoid colon (S). Bladder (B) is labeled for reference.

On examination in the emergency department, the patient's vitals were unstable with hypotension, tachycardia, and fever. On physical examination, the patient had normoactive bowel sounds, no dullness or hyperresonance to percussion, and suprapubic fullness suggestive of bladder distention, but denied tenderness to palpation in the suprapubic area as well as the four abdominal quadrants. No other masses or organomegaly were palpated. Lab work showed a white blood cell count of 24×10^9^/L (reference range: 4.5-11×10^9^/L) and low albumin of 2.4 g/dL (reference range: 3.4-5.4 g/dL) suggestive of malnutrition and a hemoglobin of 11.1 g/dL (female reference range: 12-16 g/dL). She was started on intravenous fluids, broad-spectrum antibiotics, and total parenteral nutrition. A computed tomography (CT) scan with intravenous contrast of the abdomen and pelvis showed inflammation of the sigmoid colon compatible with diverticulitis/colitis. A fistulous tract was visualized between the sigmoid colon and the fundus of the uterus (Figure 1 and Figure 2). There was fluid distending the cervix and lower canal, and air was noted within the uterus. Contrast was present in the colon and small bowel with no evidence of obstruction. At this point, a preliminary diagnosis of diverticulitis with colouterine fistula was made. The patient was monitored for five days at the outside institution while awaiting transfer to a higher level of care for definitive treatment.

The patient was admitted to our facility on day 6 under the gynecologic oncology service. General surgery was also consulted at this time. The patient's most recent labs showed significant improvement of infection with a white blood cell count of 5.5×10^9^/L (reference range: 4.5-11×10^9^/L), but worsened anemia with a hemoglobin of 9.3 g/dL (female reference range: 12-16 g/dL). The tumor markers cancer antigen 125 (CA-125) and carcinoembryonic antigen (CEA) were evaluated, and both were found to be within the normal range, with CA-125 being 17.4 U/mL (reference range: 0-35 U/mL) and CEA being 1.7 ng/mL (reference range: 0-2.5 ng/mL). On day 7, the patient had a pelvic exam and pap smear performed. On examination, the vulva and vagina showed no visible abnormality, there was no stool in the vaginal vault, and the cervix was smooth with no lesions. There was no visible fistula on the speculum exam, nor was there any palpable defect with digital vaginal examination. The uterus felt enlarged and somewhat displaced to the right on the bimanual exam. A pap smear was sent for cytology, and the following day, the report came back negative for intraepithelial lesions or malignancy; however, the endocervical component could not be identified due to severe atrophy. At this time, a multidisciplinary operative plan between gynecologic oncology and general surgery was developed. Risks, benefits, and alternatives were discussed with the patient in great detail including the risks of bleeding, infection, and damage to surrounding structures such as the bowel, bladder, and ureters. The patient wished to proceed and was scheduled for an exploratory laparotomy, total abdominal hysterectomy with bilateral salpingo-oophorectomy, and colon resection with primary anastomosis versus colostomy.

On day 9, the patient was taken to the operating room, a Foley catheter was placed, and a laparotomy was performed with a vertical midline incision. The entire small bowel was examined and appeared healthy. The appendix was identified, as well as an extensive mass consisting of intra-abdominal adhesions. There were dense adhesions found from the tip of the appendix to the rectosigmoid colon and the colouterine fistula, which was visualized at this time. Dissection was carried down to the base of the appendix, which was subsequently divided with a powered stapler at its base and removed to be sent for analysis. The sigmoid colon was transected where it appeared free of disease, which was a few centimeters proximally to where it entered the fistula. Next, bilateral ureterolysis was performed from the pelvic brim to the insertion of the bladder, and vessel loops were placed around the ureters. This was necessary secondary to retroperitoneal fibrosis that was very extreme. Dissection was continued beneath the colon, and pelvic washings were performed to be sent for analysis. The ovarian vessels were cauterized with the LigaSure vessel sealing device, and the bladder was dissected to below the level of the external cervical os. The uterine vessels were then cauterized bilaterally with the LigaSure vessel sealing device, and the parametrial tissue was dissected to below the level of the external cervical os. This area had extremely severe inflammation, and during dissection, the left ureter was injured at the level where it inserts into the bladder which became apparent later in the case. A colpotomy was performed on the anterior vagina and extended all the way around the upper portion of the vagina. The mesorectum was then dissected circumferentially and stapled across at the base, creating a rectal stump. The rectosigmoid colon/uterus/cervix/bilateral adnexa was removed en bloc and sent for analysis. The endometrium was sent for frozen section, which was found to be benign. The vaginal cuff was closed, and it was at this point that the ureteral injury was noted. Cystoscopy was performed, and the left ureter was reimplanted by the on-call urologist with subsequent bilateral ureteral stent placement and bladder decompression with a Foley catheter. At this point, the decision was made to proceed with end colostomy. The abdominal wall was closed, and a tension-free end colostomy was fashioned on the left side of the abdomen on the preoperatively marked site. The stoma was well-perfused, and the colostomy appliance was placed. The midline incision was dressed, and the patient was extubated and taken to the post-anesthesia care unit in stable condition. The patient had an uneventful recovery and was discharged to home healthcare on postoperative day 7.

**Figure 3:**
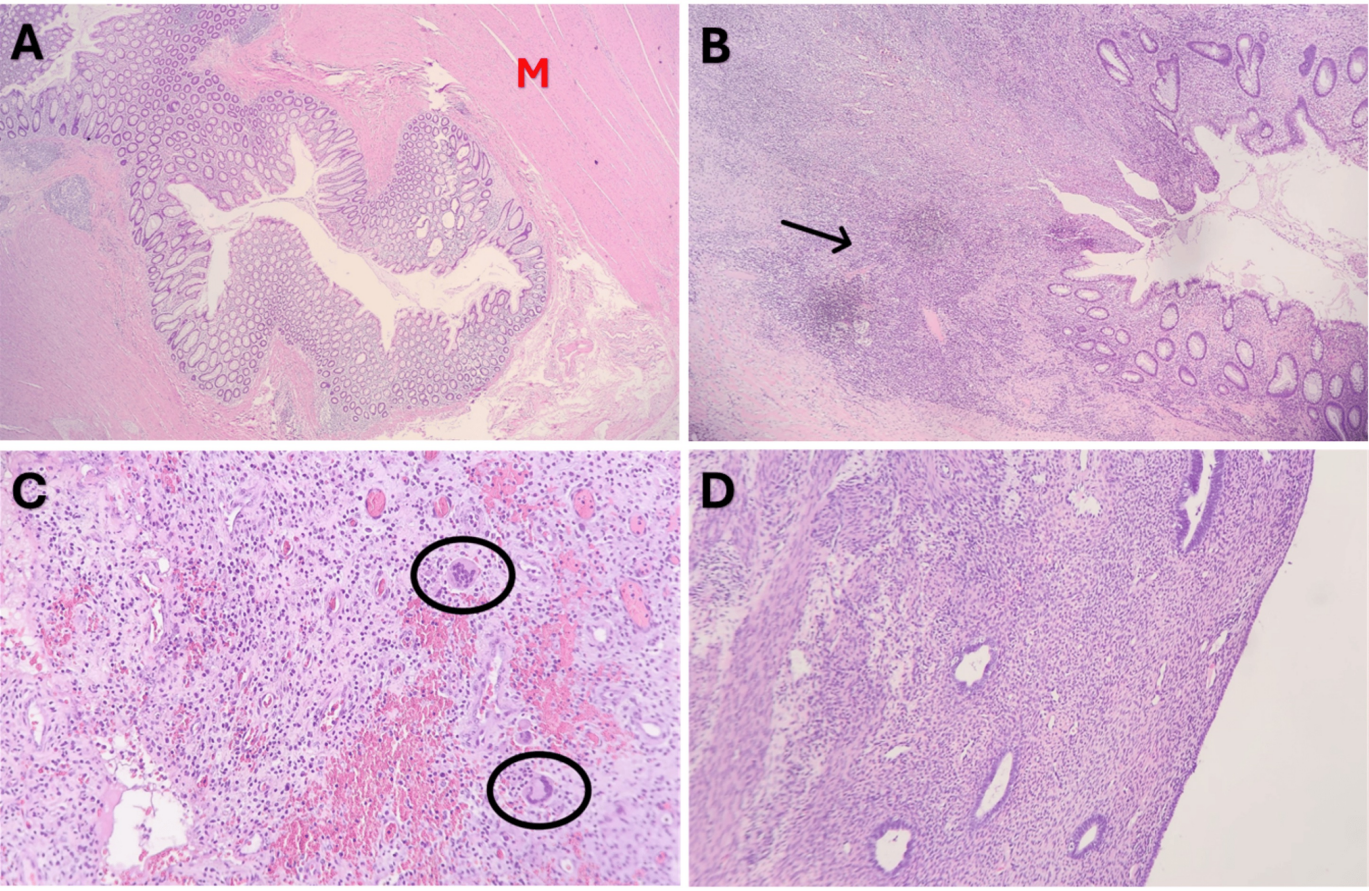
Histopathologic findings from surgically resected specimen. The sigmoid colon demonstrating diverticular disease as seen in (A) showing diverticula protruding through the muscular layer (M) of the sigmoid colon and (B) showing acute diverticular rupture with reactive atypia (black arrow). The specimen demonstrated findings compatible with colouterine fistula as seen in (C) showing the uterine wall with fibrosis, acute and chronic inflammation, and multinucleated giant cells (black circles). The absence of uterine malignancy was confirmed as seen in (D) showing benign endometrium with no significant pathologic changes.

The final histopathology report revealed the following: (a) colon with diverticular disease (Figure 3A), showing acute ruptured diverticulitis with ulceration and reactive atypia (Figure 3B), abscess formation, multiple chronically inflamed diverticula, and acute serositis and adhesions; (b) colon severely adherent to uterine and cervical walls, with fibrosis, acute and chronic inflammation, and multinucleated giant cells compatible with colouterine fistula (Figure 3C); (c) three reactive lymph nodes; (d) uterine cervix with acute on chronic cervicitis; (e) uterine body with weakly proliferative endometrium (Figure 3D) and acute and chronic inflammation of the outer half of the uterine wall; (f) bilateral ovaries with the usual physiologic changes, chronic inflammation, and surface adhesions; (g) bilateral fallopian tubes with edematous changes and chronic inflammation and adhesions; (h) appendix with mild acute appendicitis and hyperplastic polyp and benign mesothelial cysts; and (i) pelvic washings negative for malignant cells and positive for reactive mesothelial cells, lymphocytes, and histiocytes.The report confirmed the diagnosis of colouterine fistula secondary to diverticular disease and ruled out malignancy.

## Discussion

Studies show that diverticulosis is the most common colonoscopic finding among those at routine risk [14]. It was also found that the prevalence of diverticulosis steadily increased with age, rising from 32.6% in those aged 50-59 to 71.4% in those aged greater than 80 [14]. There are many complications of diverticular disease; however, fistula formation is uncommon and research reports that only 20.4% of surgically treated diverticular disease patients present with an internal fistula [3]. Of those, the most common type is colovesical comprising 65%. The next is colovaginal comprising 25%, followed by coloenteric found in 6.5% and colouterine found in only 3% [3]. Fistulas to other unusual adjacent organs have been found and documented such as the seminal vesicles [15]. In our case, there was subsequent appendicitis due to the extensive inflammatory disease causing dense adhesions to the appendix. However, colouterine fistulas secondary to diverticular disease are so rare that only a few dozen case reports can be found in the literature worldwide [5-13].

The pathophysiology behind fistula formations is the transmural destruction of the walls of two epithelialized surfaces in close proximity, resulting in a connection between the two. The thickness of the uterine myometrium affords protection against this, and it is speculated to be the reason that so few colouterine fistulas have been documented [3]. There are a few theories as to how the formation of fistulas between the colon and the uterus develops: one is that inflammatory adhesion of the bowel wall to the uterus results in necrosis of the uterine myometrium that allows for fistula formation [3] and another is that localized perforations of diverticula result in pericolic abscess, which can erode into the viscera of the uterus [3]. However, colouterine fistulas more commonly develop secondary to other pathology rather than diverticular disease, those being malignancies, radiation, and obstetric and instrumentation trauma [3]. This explains why a thorough investigation consisting of extensive history-taking, physical examination, and imaging studies is required in those suspected of having a colouterine fistula.

Most case reports show that colouterine fistulas present with similar symptoms to colovaginal fistulas; the latter, however, almost always occurs following hysterectomy [4]. Patients usually present with a history of vaginal discharge that is foul-smelling, purulent, or hemorrhagic to feculent [4]. Patients may also present with lower abdominal pain and alterations in bowel habits that align with symptoms of diverticulitis [4]. Presentation of symptoms can vary widely however, with some being asymptomatic with an unremarkable physical exam and others being in florid sepsis with an obvious pelvic mass [4]. However, a vaginal speculum exam in a colovaginal fistula will demonstrate drainage from an opening at the apex of the vagina, whereas a colouterine fistula may have a completely normal pelvic exam as seen in our patient [4].

Our patient presented with signs of sepsis, complaining of hot flashes and night sweats for the past month with new-onset diarrhea and urinary retention. She denied abdominal pain, vaginal discharge or bleeding, hematuria, or melena. She noted there had been no previous fluctuation in her bowel habits and denied any history or symptoms of diverticular disease. Her physical examination was relatively normal aside from suprapubic fullness. Her pelvic exam showed no discharge in the vaginal vault or at the cervical os, but the uterus was felt to be enlarged and somewhat displaced to the right. Given the patient's family history of cancer, as well as her age and absence of previous surgeries or known diverticular disease, malignancy was a top consideration. There was no clinical sign of internal fistula on the initial presentation; however, the preliminary diagnosis was made following the CT scan of the abdomen and pelvis performed in the emergency department, which was done to evaluate her urinary retention and diarrhea. The diagnosis was then confirmed intraoperatively and on histopathology.

The definitive treatment for colouterine fistula is surgery [5]. There are various options when it comes to the management of the uterus, which depend on the clinical suspicion of malignancy in the patient. In cases where malignant etiology cannot be excluded or when the colon and the uterus cannot be separated due to chronic inflammation, an en bloc hysterectomy is recommended [5]. However, if the suspicion of malignant etiology is low and the colon can be dissected from the uterus, then the fistulous tract can be severed, the opening can be closed, and the uterine cavity can be drained via the os [5]. This may be a more attractive option in younger patients who wish to preserve fertility. The management of the origin of the fistula, usually the sigmoid colon, is relatively straightforward given the new guidelines from the World Society for Emergency Surgery. In 2020, they released an update that recommends a sigmoidectomy with end colostomy rather than primary anastomosis in critically ill patients, in select patients with multiple comorbidities, and in those with diverticular perforation [16].

In this case, because of the patient's family history and age, a malignant etiology of the uterus could not be ruled out. The patient also stated that she would prefer a more aggressive approach and wanted the peace of mind that she did not have cancer. Because of these reasons, the dissection was conducted as an en bloc resection of the rectosigmoid colon, uterus, cervix, and bilateral adnexa. This approach allowed for the minimization of the risk of leaving behind a residual tumor had the final histopathologic report shown a malignancy and lower chances of residual infection and obviated the need to drain the contents of the uterus postoperatively, which avoids prolonging the patient's stay and decreases the risk for continued sepsis. In our patient, because of the presence of profound inflammatory changes in the rectal stump, in addition to the overall poor nutritional status, the surgeons decided against primary anastomosis. An end colostomy was created with plans for a reversal at a later date in order to allow time for the inflammation to subside and maximize the opportunity of a full recovery.

## Conclusions

Colonic diverticular disease resulting in colouterine fistulas as a complication is extremely rare with few documented cases. Colouterine fistulas secondary to other etiologies such as malignancy must be excluded through a thorough clinical investigation to develop the appropriate treatment plan for the patient. The surgical management must be tailored to the patient's individual circumstances and clinical picture. Although the presence of a colouterine fistula as a sequela of diverticulitis is rare, the prognosis has been reported to be favorable. The highlights of this case are being presented to add to the limited reports in the literature on this rare complication, which was successfully managed with surgical resection, and to spread awareness of the atypical presentation in hopes of assisting physicians encountering similar cases in the future.
